# Empirical transarterial embolization in angiographically negative lower gastrointestinal bleeding using vessel tracking and 3D navigation tools: report of 2 patients

**DOI:** 10.1186/s42155-023-00372-z

**Published:** 2023-04-20

**Authors:** Laurens Hermie, Luc Defreyne

**Affiliations:** Department of Vascular and Interventional Radiology, Ghent University Hospital, Corneel Heymanslaan 10, Ghent, 9000 Belgium

**Keywords:** Gastrointestinal hemorrhage, Lower gastrointestinal tract, Embolization, therapeutic, Cone-beam computed tomography, Arterial intervention

## Abstract

**Background:**

Recently, an empiric Cone-beam Computed Tomography (CBCT)-guided transarterial embolization (TAE) technique has been investigated for lower gastrointestinal bleeding (LGIB). Although this empirical strategy reduced the rate of rebleeding in hemodynamically unstable patients compared to a ‘wait and see’ strategy, the specified technique is challenging and time-consuming.

**Case presentation:**

We present two methods to perform a prompt empiric TAE in LGIB when catheter angiography is negative. Based on the pre-procedural Computed Tomography Angiography bleeding site and using vessel detection and navigation software tools that are integrated in contemporary angiosuites, the culprit bleeding artery could be targeted with only one selective intraprocedural CBCT acquisition.

**Conclusion:**

The proposed techniques are promising to reduce procedure time and facilitate the implementation of empiric CBCT-guided TAE in clinical practice when angiography is negative.

## Introduction

Empirical transarterial embolization (TAE) is a well-known strategy in interventional radiology (IR). In upper gastrointestinal bleeding, when primary endoscopic hemostasis has failed, endoscopy-directed empiric TAE of the left gastric or gastroduodenal artery is recommended if angiography is negative (Loffroy et al. 2021; Yu et al. 2021). For lower gastrointestinal bleeding (LGIB), the anatomy of the mesenteric arteries is not amenable for such a deliberate non-targeted TAE. Recently, however, a super-selective empirical TAE technique has been investigated for LGIB when catheter angiography is negative. Here, guided by the positive pre-procedural Computed Tomography Angiography (CTA) the suspected culprit bleeding vessel was tracked down using repetitive intra-procedural Cone-beam Computed Tomography (CBCT) angiograms (Hermie et al. 2021). In hemodynamically unstable LGIB, this empiric TAE strategy reduced the rebleeding rate significantly compared to a conservative “wait-and-see” management. However, the specified technique is challenging and time-consuming, so there may be some reluctance to apply it in clinical practice. In this report we present two methods to perform an empirical TAE in LGIB more easily using guidance tools that are integrated in contemporary angiosuites and that many of us already use for other intra-arterial treatments (Carrafiello et al. 2016).

## Patient 1

**Fig. 1:**
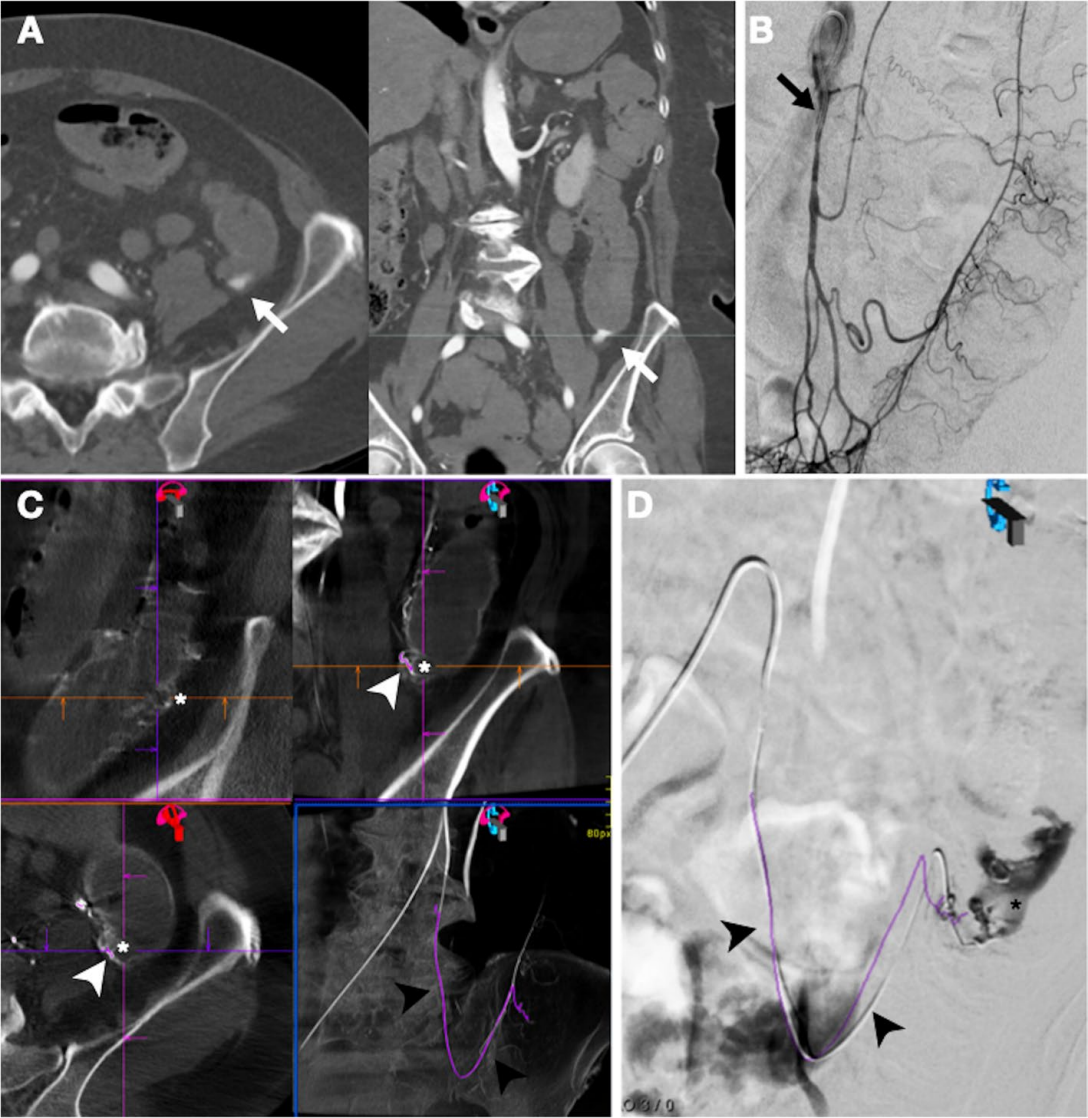
Empiric TAE approach in patient 1 **A** Pre-procedural arterial-phase CTA MPR images demonstrates an extravasation in sigmoid diverticula (white arrow). **B** No extravasation on subsequent DSA with contrast injection in the inferior mesenteric artery (black arrow). **C** Volumetric inferior mesenteric CBCT dataset displayed as MPR images in the 4D workplace. Although again no bleeding could be demonstrated, this allowed to identify the culprit diverticula (white asterisk) based on prior CTA. Then, the adjacent vas rectum (white arrowhead), presumably responsible for diverticular arterial supply and LGIB, was pointed as target for vessel detection. After also designating the catheter tip as proximal starting point, the vessel trajectory to the defined target could be identified semi-automatically (black arrowhead). **D** 3D roadmap (black arrowhead) virtually displayed on fluoroscopy for real-time guidance. After engaging the targeted vas rectum with a microcatheter, super-selective contrast injection ultimately reveals an extravasation (black asterisk)

A 76y old female patient with a hemodynamically unstable LGIB and a positive CTA, was referred to our IR department for emergent TAE after initial hemodynamic resuscitation. Despite persistent red blood per anum (RBPA) and vasopressor requirements upon arrival, subsequent inferior mesenteric Digital Subtraction Angiography (DSA) and CBCT angiography (Artis Q Angiography System; Siemens Healthineers) failed to demonstrate active bleeding. Because of hemodynamic instability and risk of bleeding recurrence, an empirical TAE (illustrated in Fig. 1) was pursued. Therefore, based on the pre-procedural CTA contrast extravasation, the suspected bleeding diverticula was exactly defined on the inferior mesenteric CBCT angiogram. This we did by comparing the CTA multiplanar reconstruction (MRP) images with equivalent MPR images of the volumetric inferior mesenteric CBCT dataset. Then, the suspected bleeding vas rectum (closest to the identified bleeding site) could be manually designated as target in the 4D workplace. After also indicating the catheter tip as the starting position, the vessel detection software (syngo Embolization Guidance, Siemens Healthineers) semi-automatically identified the vascular pathway between the selected points. By virtually displaying the segmented 3D roadmap on fluoroscopy, a 2.7 F microcatheter could be accurately navigated into the targeted vas rectum. Super-selective contrast injection did eventually show extravasation. After TAE of the targeted vas rectum using N-butyl cyanoacrylate (NBCA) glue, the patient had a clinically reassuring course with no recurrent bleeding or ischemic complications.

## Patient 2

**Fig. 2:**
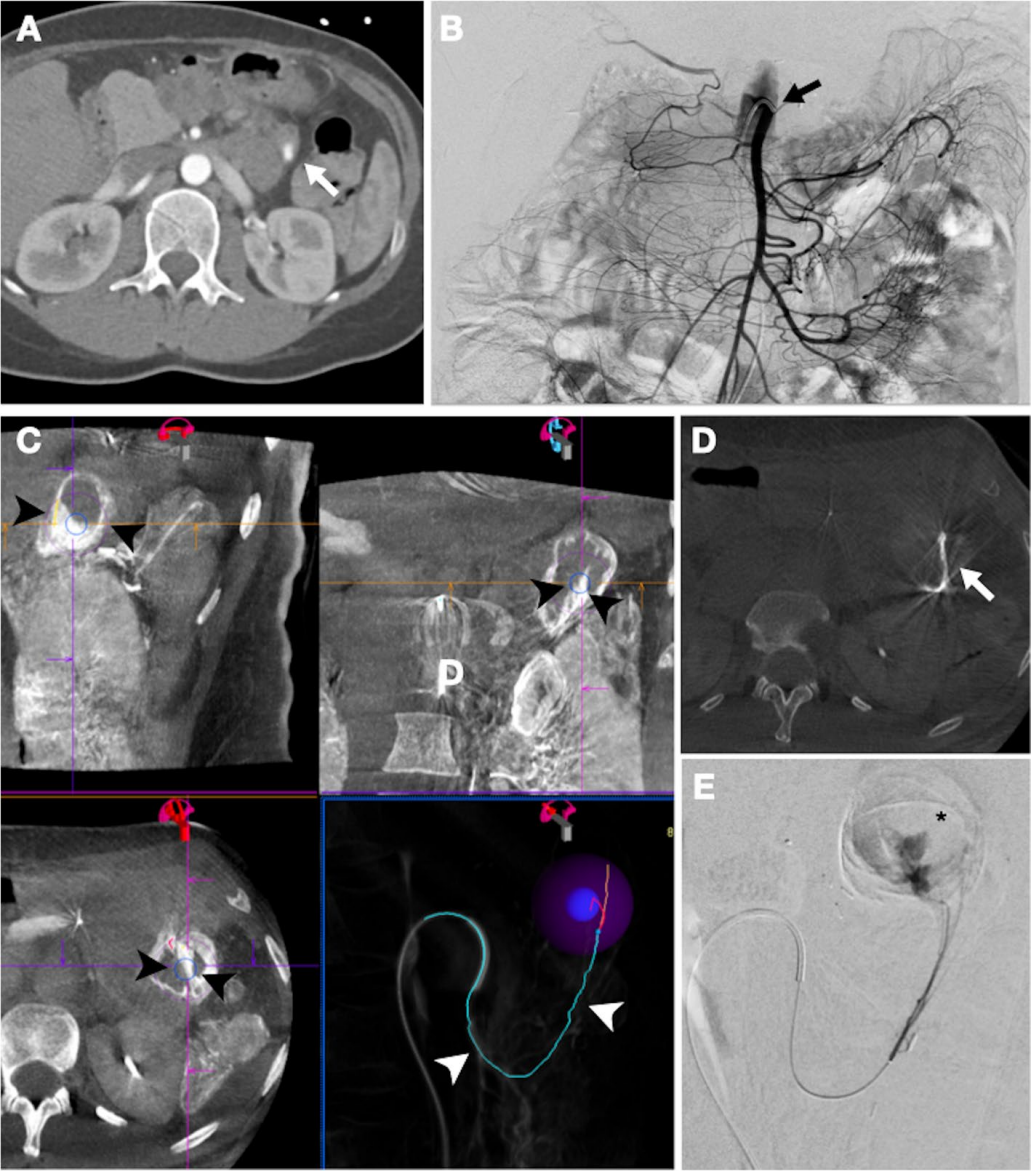
Empiric TAE approach in patient 2 **A** Pre-procedural arterial-phase CTA displays a contrast extravasation proximally in the Roux-en Y jejunal loop (white arrow). **B** No bleeding focus on subsequent DSA with contrast injection in the superior mesenteric artery (black arrow). **C** Superior mesenteric CBCT MPR images in the 4D workplace. Here, using prior CTA bleeding focus, mucosal enhancement in the suspected culprit jejunal bowel segment was highlighted as ROI (black arrowhead) for automatic vessel detection. The resulting 3D vessel segmentation (white arrowhead) was used for catheter navigation. **D** A CBCT acquisition with super-selective contrast injection in the engaged artery confirmed correct targeting trough the mucosal enhancement (white arrow) overlapping the previously decided ROI (intended bleeding site). **E** Contrast injection after selecting the targeted artery eventually showed an extravasation (black asterisk)

38y old female patient with history of a lateral pancreatojejunostomy after pancreatic trauma was admitted because of recurrent RBPA and one-time hematemesis. Initial upper gastrointestinal endoscopy and CTA could not identify an active bleeding focus. However, because of progressive hemodynamic instability, the patient was transferred the following day to our tertiary referral hospital. A new CTA now did reveal an active bleeding proximally in the Roux-en Y jejunal loop and the patient was immediately transferred to the angiosuite. Nevertheless, superior mesenteric DSA and CBCT failed to identify a bleeding focus. Regarding inaccessibility for endoscopy, empirical TAE (illustrated in Fig. 2) was considered the most appropriate strategy to prevent rebleeding. Using the CTA bleeding focus, the targeted jejunal bowel segment could be identified on comparable superior mesenteric CBCT MPR images. Then, the mucosal enhancement in the depicted jejunal bowel segment (as close as possible to the expected bleeding site) was highlighted as region of interest (ROI). Here, automatic vessel tree detection software (syngo Embolization Guidance, Siemens Healthineers) identified the arterial supply to the ROI. After visual review and affirmation of the vessel tree segmentation, a 2.0 F microcatheter was advanced along the 3D roadmap to the target site. Then, a super-selective CBCT acquisition confirmed correct catheter placement through the mucosal enhancement overlapping the previously decided ROI. Eventually, after additional manual contrast injection in the engaged artery revealed an extravasation, the vascular supply was super-selectively embolized with NBCA glue. The patient recovered well after the procedure and no recurrent bleeding or complications occurred.

## Discussion

Recent guidelines emphasize the importance of CTA and TAE in the management of active or hemodynamically unstable LGIB (Triantafyllou et al. 2021; Oakland et al. 2019; Strate and Gralnek 2016). While a negative CTA usually precludes immediate intervention, a positive CTA is considered as an indication for emergency catheter angiography (Chan et al. 2015). Then, if extravasation is demonstrated on angiography, targeted TAE can be undertaken with a high technical success rate (Hermie et al. 2021; Kwon et al. 2019). However, in as many as half of the patients, the bleeding is not identified on angiography and thus no TAE is performed (Hermie et al. 2021; Maleux et al. 2009). We demonstrated in both presented patients how one can still attempt a TAE in this unfavourable situation with high risk of rebleeding. In essence, after determination of the CTA bleeding site within the inferior or superior mesenteric vasculature, using modern vessel tracking and navigation tools one can target the suspected vessel tree with only a single selective CBCT acquisition. In contrast, an average of 4.0 CBCTs were required to perform a TAE in the original empiric CBCT-guided technique (Hermie et al. 2021). Although embolization in both patients was not strictly empirical, the extravasation appeared only after targeting the suspected vas rectum based on prior positive CTA. Therefore, empirical TAE would have been identical even without the appearance of extravasation. Conversely, it ultimately confirms the effectiveness of this empirical approach. Even if the vessel tracking software would identify multiple arterial supply to the ROI, this does not preclude an empirical embolization. To overcome ischemic complications, TAE of up to 3 vasa recta has been shown to be acceptable (Kwon et al. 2019). Nevertheless, it is imperative that the delineation of the ROI is performed precisely, and that adjacent non-targeted bowl segments are withheld from the designated area. Therefore, after engaging the targeted vasculature, an additional super-selective CBCT angiogram is still recommended to demonstrate precise overlap of the mucosal enhancement with the pre-procedural CTA bleeding site.

## Conclusion

The use of vessel detection and navigation software could be promising to reduce procedure time and facilitate the implementation of empirical TAE in clinical practice. However, safety and efficacy of this approach should be verified on a larger scale.

## Data Availability

Data sharing is not applicable to this article as no datasets were generated or analysed during the current study.

## Abbreviations

CBCT: Cone-beam computed tomography
CTA: Computed tomography angiography
IR: Interventional radiology
TAE: Transarterial embolization
LGIB: Lower gastrointestinal bleeding
MPR: Multiplanar reconstruction
NBCA: N-butyl cyanoacrylate (NBCA)
ROI: Region of interest.
